# The role of mesenchymal stromal cells in immune modulation of COVID-19: focus on cytokine storm

**DOI:** 10.1186/s13287-020-01849-7

**Published:** 2020-09-18

**Authors:** Maria Kavianpour, Mahshid Saleh, Javad Verdi

**Affiliations:** 1 Department of Tissue Engineering and Applied Cell Sciences, Faculty of Advanced Technologies in Medicine, Tehran University of Medical Sciences, Tehran, Iran; 2 Cell-Based Therapies Research Center, Digestive Disease Research Institute, Tehran University of Medical Sciences, Tehran, Iran

**Keywords:** Mesenchymal stromal cells, COVID-19, Immune regulatory, Cytokine storm

## Abstract

The outbreak of coronavirus disease 2019 (COVID-19) pandemic is quickly spreading all over the world. This virus, which is called SARS-CoV-2, has infected tens of thousands of people. Based on symptoms, the pathogenesis of acute respiratory illness is responsible for highly homogenous coronaviruses as well as other pathogens. Evidence suggests that high inflammation rates, oxidation, and overwhelming immune response probably contribute to pathology of COVID-19. COVID-19 causes cytokine storm, which subsequently leads to acute respiratory distress syndrome (ARDS), often ending up in the death of patients. Mesenchymal stem cells (MSCs) are multipotential stem cells that are recognized via self-renewal capacity, generation of clonal populations, and multilineage differentiation. MSCs are present in nearly all tissues of the body, playing an essential role in repair and generation of tissues. Furthermore, MSCs have broad immunoregulatory properties through the interaction of immune cells in both innate and adaptive immune systems, leading to immunosuppression of many effector activities. MSCs can reduce the cytokine storm produced by coronavirus infection. In a number of studies, the administration of these cells has been beneficial for COVID-19 patients. Also, MSCs may be able to improve pulmonary fibrosis and lung function. In this review, we will review the newest research findings regarding MSC-based immunomodulation in patients with COVID-19.

## Introduction

The city of Wuhan was the origin of coronavirus disease (COVID-19), a severe acute respiratory syndrome with SARS-CoV-2 as its causative agent. Presently, COVID-19 infection has spread to all continents of the world [1]. Due to unknown reasons, COVID-19 infection has been widely distributed in various geographical regions with high population densities [2]. Moreover, the profile of symptoms and severity of COVID-19 infection show extensive variation in different parts of the world [3]. Worldwide assessments suggest that only 3.4% of those infected with SARS-CoV-2 have perished as a result of COVID-19, which also shows high difference in various parts of the world [4].

Constant fever, non-productive cough, dyspnea, myalgia, fatigue, normal or reduced WBC counts, hyperferritinemia, and radiographic evidence of pneumonia are among the clinical signs of patients with COVID-19, which are similar to the symptoms of infection by other members of this family, namely SARS-CoV and the Middle East respiratory syndrome-related coronavirus (MERS-CoV) [5–7]. The mortality rate of the new coronavirus, known as SARS-CoV-2, is high because of insufficient knowledge about the pathogenesis of COVID-19, and no specific treatment has been recognized for it [8]. On the other hand, the response to COVID-19 infection can be overwhelmed in many patients. When SARS-CoV-2 enters into the lungs, it unleashes an immune response, attracting immune cells to the region attacked by the virus and resulting in localized inflammation [9]. In some cases, excessive or unchecked levels of cytokines are released that can be fatal due to an overreaction of the immune system, which is referred to as a cytokine storm [10]. The cytokine storm can trigger organ injury and cause edema, gas exchange dysfunction, acute respiratory distress syndrome (ARDS), acute cardiac injury, and secondary infection, which can be potentially fatal [11].

Consequently, the inhibition of cytokine storm is a main factor in the treatment of patients who are infected with SARS-CoV-2. Currently, available therapies for COVID-19 include non-specific antiviral drugs, antibiotics used for the treatment of secondary bacterial infections, sepsis, and reduction of inflammation [12]. A large number of anti-inflammatory medications have been developed, including NSAIDs, glucocorticoids, chloroquine/hydroxychloroquine, antagonists of inflammatory cytokines (such as IL-6R monoclonal antibodies, TNF inhibitors, IL-1 antagonists), and Janus kinase JAK inhibitors [13, 14]. However, in severe cases of ARDS, it is a difficult task to treat the cytokine storm induced by the virus. The findings suggest that stem cell-based therapy is applicable to treat infected patients.

### Mesenchymal stromal cells and their features

Mesenchymal stromal cells (MSCs) are the cells with the unique ability to exert suppressive and regulatory effects on the immune system [15]. MSCs have been the focus of research because evidence has indicated that MSCs are able to migrate to and return from damaged tissues, exercise potent anti-inflammatory and immune regulatory activities, support the regeneration and repair of tissues, resist against apoptosis, inhibit tissue fibrosis, and decrease tissue injury [16]. MScs are able to migrate to site of lesion and differentiate into tissue-specific active cells such as lung, smooth muscle, and nerve cells [17]. Following intravenous or intra-arterial infusion of MSCs, these cells are primarily trapped in capillary beds of the liver and lungs [18]. MSC homing processes are not fully realized but are known to involve a variety of molecules such as chemokine receptors, including CCR2, CCR4, CCR7, CCR10, CXCR5, CXCR6, and CXCR4, adhesion proteins, and matrix metalloproteinase (MMPs), namely molecules also implicated in the well-known process of leukocyte extravasation [19, 20]. Hypoxia and inflammation are frequent indications of an injured tissue capable of affecting paracrine features of MSCs, which are mainly mediated via VEGF, FGF2, IGF-1, and HGF [21].

When MSCs are trapped in the lungs, a wide range of soluble mediators are secreted by them, including antimicrobial peptides, anti-inflammatory cytokines, extracellular vesicles, and angiogenic growth factors [22]. The release pattern of anti-inflammatory mediators is unique to the inflammatory lung environment, which is adjusted by differential damage and pathogen-associated molecular receptors that are expressed on MSCs [23], namely TLRs (toll-like receptors). As for COVID-19, TLRs are stimulated by viral unmethylated CpF-DNA (TLR9) as well as viral RNA (TLR3), leading to sequential cellular signaling pathways and the activation of MSCs [24].

On the other hand, inflammation leads to nuclear factor-kappa B (NF-κB) and c-Jun NH2-terminal kinase (JNK) signaling, which is also controlled through the factors secreted by MSCs. In addition, lung damage improves during the response of MSCs to oxidative stress, cytoprotection, and phosphoinositide 3-kinase/protein kinase B (P13K / Akt) signaling pathway [25]. Administration of BM-MSCs alleviated lung injury in a preclinical study via potentiating the PI3K/Akt signaling pathway [26, 27].

For example, the release of IL-1ra through MSCs inhibits IL-α/β activity via generating TSG-6, which is followed by the downregulation of NF-κB signaling and reduced production of inflammatory cytokines. Secretion of prostaglandin E2 (PGE2) is another efficient way to decrease inflammation by MSCs, which is a function of IL-10 production as a strong anti-inflammatory cytokine. Khakoo et al. showed that MSCs prevent PKB signaling of target cells via a contact-dependent way [28].

MSCs encounter a complex setting specified by various chemical and physical stimuli while moving toward an injured tissue and the microenvironment impacts MSCs’ behavior [29]. MSCs are able to release many types of cytokines through paracrine release or direct interaction with immune cells, which leads to immunomodulation [30]. These cells have the capacity to interact with immune cells in innate and adaptive immune systems [31]. Besides, MSC-mediated immunosuppression depends on the combined reaction of chemokines, inflammatory cytokines, and effector factors, as along with the microenvironment and the rate of inflammatory stimulus [32]. Owing to their powerful immunomodulatory ability, MSCs might have beneficial effects for preventing or attenuating the cytokine storm of SARS-CoV-2 infection [33, 34]. This paper tries to explain the significant role of MSCs in secreting important factors for immune regulation in COVID-19.

### The SARS-CoV-2 infection and cytokine storm

**Fig. 1:**
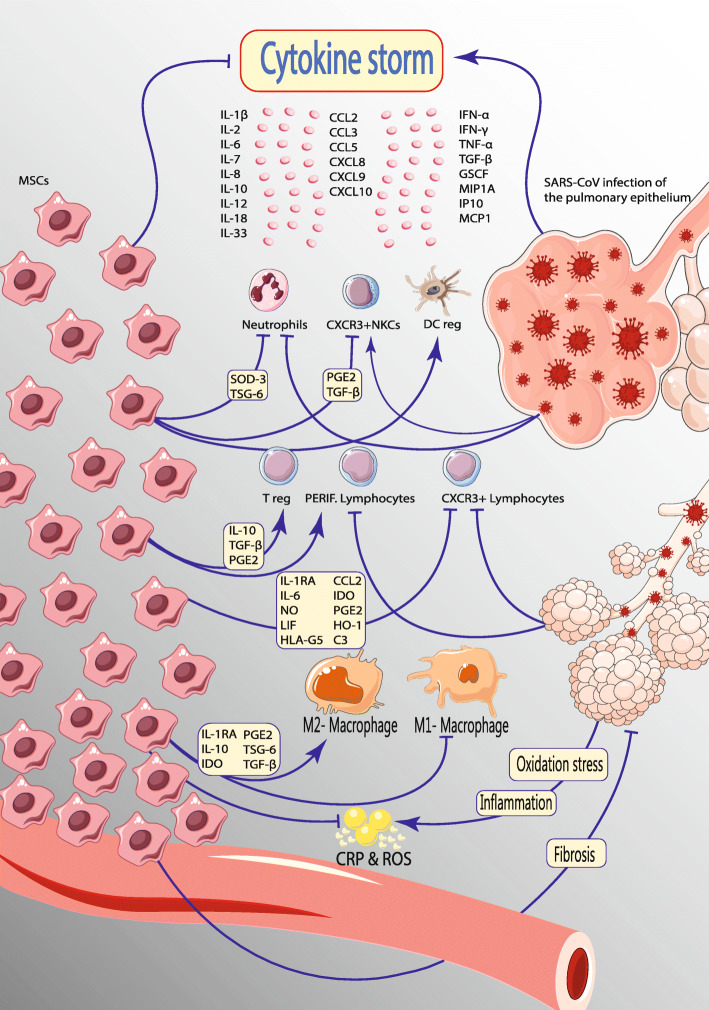
Immunomodulation effect of mesenchymal stem cells on cytokines storm led by COVID-19. When SARS-CoV-2 enters the lungs, it attracts immune cells to infection areas and localizes inflammation. The lethal unchecked systemic inflammatory response is caused by the secretion of large levels of pro-inflammatory cytokines such as interleukin, interferons, chemokines, and other factors by immune effector cells in this infection. After MSC therapy, these cells reach the lung tissue and secrete factors that can modulate the immune system; they also can prevent ROS and even fibrosis of the lung tissue. Abbreviation: ARDS: acute respiratory distress syndrome, COVID-19: coronavirus disease 2019, CCL: chemokine (C-C motif) ligand, CXCL: chemokine (C-X-C motif) ligand, C3: Complement component 3, CRP: C-reactive protein, DC reg: regulatory dendritic cells, GSCF: granulocyte colony-stimulating factor, HO-1: Heme oxygenase-1, HLA-G5: human leukocyte antigen-G, IL: interleukin, IFN: interferon, IP10: IFN-γ-Inducible Protein 10, IL-1RA: interleukin-1 receptor antagonist, LIF: leukemia inhibitory factor, IDO: Indoleamine 2,3-dioxygenase, MSCs: mesenchymal stem cells, MIP1A: Macrophage Inflammatory Protein 1 Alpha, MCP1: *monocyte chemoattractant protein 1*, NKCs: natural killer cells, NO: nitric oxide, PERIF: peripheral, PGE2: Prostaglandin E_2_, ROS: *reactive oxygen species,* SARS-CoV: severe acute respiratory-associated coronavirus, SOD-3: *superoxide dismutase*, TSG-6: TNFα-stimulated gene-6, TGF-β: transforming growth factor, Treg: regulatory T

ARDS caused by cytokine storm is the main mortality factor in COVID-19 [35]. The lethally uncontrolled systemic inflammatory response is stimulated by the secretion of a large number of pro-inflammatory cytokines such as interleukin (IL)-1β, IL-2, IL-6, IL-7, IL-12, IL-18, IL-33, interferon (IFN)-α, IFN-γ, tumor necrosis factor-α (TNFα), granulocyte colony-stimulating factor (GSCF), interferon-γ inducible protein 10 (IP10), monocyte chemoattractant protein 1 (MCP1), macrophage inflammatory protein 1-α (MIP1A), and transforming growth factor-beta (TGF-β) such as chemokines by immune effector cells within coronavirus infection (Fig. 1) [36–38].

Huang et al. reported the level of inflammatory factors among patients with COVID-19. They measured cytokines of patients with COVID-19 and indicated increasing levels of IL-1B, IL-1RA, IL-7, IL-8, IL-9, IL-10, fibroblast growth factor (FGF), granulocyte-macrophage colony-stimulating factor (GM-CSF), IFN-γ, G-CSF, IP10, MCP1, MIP1A, PDGF, TNFα, and vascular endothelial growth factor (VEGF) in their specimens, among which TNFα levels were higher in patients with severe disease. Remarkably, no significant difference was observed in serum IL-6 levels between ICU and non-ICU admitted patients [8]. Nevertheless, in a retrospective, multicenter cohort study, the same research group reported a significant elevation of IL-6 levels in patients not surviving COVID-19 as compared with survivors [39]. Several other reports have also confirmed increasing IL-6 levels among critically ill COVID-19 patients [24, 40]. Moreover, the result of another study demonstrated that a majority of severe COVID-19 patients in ICU had persistently elevated levels of ESR and CRP, as well as high levels of IL-6, TNFα, IL-1β, IL-8, and IL2R, and experienced ARDS, hypercoagulation, and disseminated intravascular coagulation (DIC) [13].

The cytokine storm was followed by ARDS and multiple organ failure, which causes death in severe cases of COVID-19. For example, the findings of Huang et al. showed that out of 41 infected patients who were admitted in the early stages, 6 patients died as a result of ARDS [8]. Like common acute viral infections, both humoral and cellular immunity are activated in COVID-19. Therefore, inhibition of cytokine storm may be the key to the treatment of COVID-19 patients.

### Immunomodulatory effects of MSCs

MSCs show remarkable immunomodulatory capacity and are implicated in both innate and adaptive immune systems. Former investigations on immune regulation of MSCs have concentrated on interactions of MSCs and B lymphocytes, natural killer (NK) cells, and dendritic cells (DC) [41]. Lately, the application of MSCs in repairing damaged tissue and adjustment of inflammatory reactions have become noticed considering macrophage and T lymphocyte regulation (Fig. 1) [16]. Interaction mechanisms have been shown to be dependent upon cell-cell contact along with the release of soluble immune factors to induce MSC-regulated immunosuppression [42]. The cells that express immunosuppressive ligands like programmed death-ligand 1 (PD-L1) and Fas ligand (Fas-L) on their surface bind receptors present on the surface of immune cells, which leads to loss of function in immune cells [43, 44].

Several studies have revealed that the anti-inflammatory effect of MSCs can alleviate virus-induced lung injury and mortality in mice [45, 46]. Research has indicated that MSCs are able to significantly reduce acute lung injury by H9N2 and H5N1 viruses in mice by decreasing levels of pro-inflammatory cytokines and chemokines as well as diminishing the recruitment of inflammatory cells into the lungs [47, 48]. Applying MSCs to interfere in endotoxin (LPS)-induced acute lung injury of mice proved that MSCs can remarkably lead to reduction of inflammatory cell infiltration in lung tissue, alleviate inflammation, and improve the lung tissue from endotoxin-induced damage [49, 50].

Intravenous infusion of MSCs normally results in their accumulation within lungs, whereby they secrete many paracrine factors [51]. Evidence suggests that MSCs bind activated immune cells, which could keep them in close proximity and hence potentiate immunosuppressive effects [52]. Moreover, MSCs can also prevent the function of immune cells via releasing cytokines such as TGF-β, HGF, and prostaglandin E2 (PGE2), as along with other anti-inflammatory factors [53]. For example, MSCs secrete TGF-β and other factors promoting the induction of regulatory T lymphocytes (Tregs) and M2 macrophages, transmitting the immunosuppressive effects to other cells in order to activate various immunosuppressive mechanisms [54]. MSCs express TNFα-stimulated gene/protein 6 (TSG-6) that mediates the regulation of immune inflammation (Fig. 1) [55]. TSG-6 is another key factor with a crucial role in tissue repair activity of human MSCs that has been proven in mouse models of myocardial infarction, peritonitis, and acute corneal and lung injury [23, 56]. TSG-6 antagonizes the binding of CXCL8 to heparin via interaction with the GAG-binding site of CXCL8, which inhibits CXCL8-mediated chemotaxis of neutrophils. Furthermore, TSG-6 can prevent the extravasation of leukocytes (especially neutrophils and macrophages) at the inflammation site [57].

In COVID-19 cases, MSCs are able to increase the lymphocyte count and regulatory DCs to raise their antiviral characteristic which results in the decreased level of C-reactive protein and pro-inflammatory cytokines (IL-6, TNFα, IL-8, and so on) that are the main markers of inflammation and ROS to diminish the inflammation and oxidative stress [58]. On the other hand, MSCs can increase the level of IL-10 as an anti-inflammatory protein activating regulatory cells such as Tregs (Fig. 1) [59]. Therefore, MSCs play a central role in immune homeostasis by interacting with cytokines, chemokines, and cell surface molecules. Put together, all these immunomodulatory features contribute to the extensive potential of MSCs in clinical therapies.

### MSC-based therapy in COVID-19 patients

The capacity of MSCs in multilineage differentiation and immunomodulation signifies that these somatic progenitor cells are extremely versatile in many therapeutic applications [60]. In fact, as of April 2016, more than 500 MSC-related clinical trials have been recorded on the NIH Clinical Trial Database (https://clinicaltrials.gov/) [61].

The safety and efficacy of transplanted MSCs for the alleviation of inflammatory lung diseases appears to be demonstrated in experimental models [62]. MSCs have been extensively used in cell-based therapies from basic research to clinical trials [63–65]. Inflammation has been recognized to affect several morbid processes in the pulmonary system, which include obstructive diseases such as chronic obstructive pulmonary diseases (COPD) and asthma, along with restrictive diseases such as idiopathic pulmonary fibrosis (IPF) and (ARDS). The acute and chronic lung injury observed in these diseases always involves abnormal immune activity and fibrosis either as a cause or a consequence [66, 67]. Similar to most cell therapies, MSC therapy could be useful in lung disease because it has been demonstrated that many intravenously delivered MSCs (80–90%) will rapidly reach the lungs when delivered through intravenous injection [68]. Following systemic administration, a majority of MSCs lodge in the pulmonary vascular bed through unknown interactions with the capillary endothelial cells. Tracking studies using labeled MSCs demonstrate that most MSCs are cleared within 24–48 h although there can be longer persistence of them in injured or inflamed lungs [69]. Several phase I/II clinical trials have been done to determine the safety of MSC infusions in patients afflicted with ARDS. In China, Zheng et al. observed no serious adverse events associated with MSC administration in 12 patients with ARDS [70]. Besides, Wilson et al. showed that a single intravenous MSC infusion up to 10^6^ cells/kg was well tolerated in nine patients with moderate to severe ARDS [71]. Matthay and colleagues reported a prospective, double-blind, randomized clinical trial evaluating the effect of a single systemic dose of allogeneic bone marrow-derived MSCs (10^7^ cells/kg) in comparison with placebo (2:1 ratio). This research demonstrated that no hemodynamic or respiratory abnormal events associated with MSC infusion were seen over a follow-up period of 60 days and that the 28-day death rate was higher in the MSC group relative to the placebo, which was not remarkably different between the groups [72]. Furthermore, the novel avian-origin influenza A (H7N9) virus with single-stranded RNA segments and COVID-19 has comparable complications (e.g., ARDS and lung failure), as well as corresponding multi-organ dysfunction together with inflammatory lung lesions and structural damage [73, 74]. Thus, advances in finding a therapeutic approach for H7N9 infection in human beings would be essential for treating COVID-19, especially severe ARDS-induced pneumonia that has presently provoked panic in every corner of the world [8, 75, 76]. Wilson et al. [71] have recently found that the administration of allogeneic MSCs for nine patients with ARDS did not lead to any particular adverse events such as cardiac arrhythmia, hypoxemia, and ventricular tachycardia. MSCs taken from menstrual blood have recently become attractive because of easy access, high rate of proliferation, and a non-invasive procedure lacking ethical issues [77–79]. Chen et al. argue that MSCs are capable of reducing inflammatory effects and avoiding cytokine storm. MSCs are an encouraging tool for treatment of acute pneumonia for prospective clinical application [14]. A recent study in China revealed that administration of intravenous injection of MSCs significantly improved the inflammation situation in severe COVID-19 patients. Ultimately, the patients with severe COVID-19 pneumonia survived the worst condition and entered recovery. Also, they said that the level of TNFα was significantly decreased, while IL-10 increased in MSC treatment group [80].

It should be mentioned that just a few cell therapy studies for ARDS and sepsis have reached their primary goals in randomized investigations [81, 82]. Scientists approve that targeted clinical research is essential whilst the first reports of MSC use for COVID-19 over the early phase of COVID-19 breakout in China have presented valuable clues that the therapeutic measures could be rather safe and efficient [83–85]. Few conclusions can be derived from these early studies because of the low number of subjects (typically ≤ 10) and the absence of adequate control groups [58, 80, 86]. Proper design of clinical trials and the observation of quality factors such as the documentation of patients, inclusion/exclusion criteria, classification of treatment approaches, primary and secondary statistics, timing and dosing of treatments, and comedication are immediately needed [85, 87]. While preliminary results may appear to be promising, the previous failures of innovative clinical research with MSCs as well as the low number of registered MSC products should be kept in mind [88–92]. Several problems have been identified in this respect, including failed upscaling of the product to large-scale supply and the absence of translation to efficient clinical application (e.g., cell expansion from the starting material, cell viability problems after thawing, and suboptimal delivery route) [82, 93–96], which could account for the failure of different studies [88, 89]. A key issue for sustainable marketing would be the technological adequacy of the products and manufacturers in case some of the advanced phase II/III clinical studies show solid evidence supporting the approval of product in future (as discussed below) [90, 91]. The unprecedented dynamics of COVID-19 pandemic as well as the high number of deaths all over the world indicate that large-scale manufacturing and comprehensive logistic capacity are required to supply enough doses of high-quality cellular products in a reproducible and chronological manner [97]. SARS-CoV-2 enters into cells through the recognition of cellular transmembrane protease serine 2 (TMPRSS2) and angiotensin I converting enzyme 2 receptor (ACE2). ACE2 receptors have been detected in the heart (endothelium of coronary arteries, myocytes, fibroblasts, epicardial adipocytes), blood vessels (vascular endothelial and smooth cells), gut (intestinal epithelial cells), lung (tracheal and bronchial epithelial cells, type 2 pneumocytes, macrophages), kidney (luminal surface of tubular epithelial cells), brain, and testis [98]. In the human lung, the wide surface area of alveolar epithelial cells could account for the vulnerability of this organ to negative sequelae of COVID-19 infiltration. A main point is that ACE2 receptors are largely expressed in type II pneumocytes, i.e., small cylinder-shaped cells representing 5% of all pneumocytes [99] that are responsible for the generation of alveolar surfactant while acting as “stem” cells and progenitors of type I pneumocytes (95% of pneumocytes) performing gas exchanges in lungs [100–102].

SARS-Cov and SARS-CoV2 bind ACE2 receptors, leading to membrane fusion and virus penetration into the cell, thereby causing the downregulation of these receptors [98, 103]. In other words, the virus seems to enter into the cell together with the membrane receptor, which is subsequently removed from the external surface of the membrane.

The ACE2 gene lies on X chromosome and it has been shown that potential functional variants of ACE2 gene alter its transcriptional activity. Nevertheless, the pattern of population distribution influencing differential susceptibility to SARS-CoV-2 and the genetic origin of differential ACE2 expression and its functional implications among different populations are barely known [104]. Hypertension and diabetes mellitus (DM) are the most frequent comorbid conditions in COVID-19 and both are modulated by ACE2. Loss of ACE2 disturbs the balance of renin-angiotensin system, impairing vascular function and exacerbating cardiovascular complications of diabetes [105]. It appears that higher severity of COVID-19 among those suffering from high blood pressure and DM could be driven at least partially by pathological deviations from the ACE2 pathway. Accordingly, ACE2 appears to be crucial in the outcome of COVID-19as well as the role it plays in susceptibility [106].

A major function of ACE2 has also been proven in inflammatory processes [107]. Genetic deficiency of ACE2 leads to upregulated expression of cytokines, inducing vascular inflammation in ApoE knockout mouse models [108]. In a recent research, ACE2 expression was related with several immune signatures such as markers of T cells, B cells, and NK cells, as well as interferon response in various human tissues [109]. These results suggest that ACE2 not only acts as a receptor of SARS-CoV-2 but takes part in mediation of post-infection downstream processes, including inflammatory responses. Furthermore, the gene expression profile of MSCs shows that ACE2 and TMPRSS2 are not expressed in these cells, so MSCs are not infected by a coronavirus. RNA-seq survey to identify 12,500 transplanted MSC during follow-up revealed that the cells had not been differentiated and remained ACE2 negative. ACE2 expression is observed in other tissues like the heart, liver, kidney, and digestive organs [80].

Such an expression pattern explains the reason why infected ICU patients are afflicted not only with ARDS but also other complications of multiple organ dysfunction syndromes [110]. The mechanisms that account for the improvement observed following MSC infusion in COVID-19 patients seem to be indicative of the strong anti-inflammatory activity of these cells [80]. Two recent studies from China on COVID-19 patients reveal a marked reversal of symptoms even in severe and critical conditions [58, 80]. Consequently, their clinical researches recognize a new remedial strategy and the presence of natural mechanisms capable of defeating acute inflammatory pneumonia. One study was a case report of a critically ill COVID-19 patient on a ventilator with progressive disease despite undergoing intensive therapy with markers showing liver injury. The patient was treated using allogeneic human umbilical cord MSC (hUC-MSC) and three intravenous infusions of 5 × 10^7^ hUC-MSC 3 days later. Within 4 days of the first cell infusion, the patient was detached from the ventilator and was able to walk. All the parameters under study such as circulating T lymphocytes returned to normal. Low levels of lymphocytes could be due to their sequestration within the inflamed lungs and tissues. No noticeable side effects were observed [58].

The second study [80] by Leng et al. demonstrated that intravenous MSC infusion prevents the immune system from overactivation and repairs the lung microenvironment affected by SARS-CoV-2 infection even in older patients. Intravenous infusion of MSCs normally results in the accumulation of these cells in the lungs and release of several paracrine factors [111]. MSC infusion is useful especially in older people infected with SARS-CoV-2 because this group is more susceptible to pneumonia from SARS-CoV-2, resulting in severe respiratory distress and mortality because of immunosenescence [112–114]. It is proved that the intravenous infusion of MSCs is a safe and efficient orientation for treating patients infected by COVID-19 pneumonia, including older people with severe pneumonia [115].

Nowadays, several clinical trials using stem cell therapy to treat the coronavirus have been recorded from China, Iran, USA, Columbia, France, Denmark, Jordan, and Saudi Arabia, which are listed at www.clinicaltrials.gov, www.chictr.org, www.irct.ir and summarized in Table 1.

**Table 1:** Overview of planned or ongoing studies of cell therapy for the treatment of COVID-19

NO	Title and sponsor	Trial ID	Location	Design	Primary outcome	Recruitment status	Phase
1.	A Randomized, Double-Blind, Placebo-Controlled Clinical Trial to Determine the Safety and Efficacy of Hope Biosciences Allogeneic Mesenchymal Stem Cell Therapy (HB-ADMSCs) to Provide Protection Against COVID-19 Sponsor: Hope Biosciences	NCT04348435	Texas, USA	Randomized, placebo-controlled, double-blinded, clinical trial to assess effectiveness of HB-allogeneic adipose-derived mesenchymal stem cells to supply immune support against coronavirus illness.N:100.	• Incidence of hospitalization for COVID-19 • Incidence of symptoms associated with COVID-19	Enrolling by invitation April 16, 2020	Phase 2
2.	A Clinical Trial to Determine the Safety and Efficacy of Hope Biosciences Autologous Mesenchymal Stem Cell Therapy (HB-ADMSCs) to Provide Protection Against COVID-19 Sponsor: Hope Biosciences	NCT04349631	Texas, USA	Open label, single-center, and clinical trial to assess effectiveness of HB-ADMSCs to produce immune support against coronavirus illness.N:56.	• Incidence of hospitalization for COVID-19 • Incidence of symptoms associated with COVID-19	Enrolling by invitation April 16, 2020	Phase 2
3.	Natural Killer Cell (CYNK-001) Infusions in Adults With COVID-19 (CYNK-001-COVID-19) (CYNK001COVID) Sponsor: Cellularity Incorporated	NCT04365101	?	Open label, randomized, phase I can assess the safety and effectivity of multiple doses of CYNK-001 (days 1, 4, and 7) in 14 patients. Phase II can utilize a randomized, open-label design; multiple doses of CYNK-001 are compared to the control group. Up to 72 patients are enclosed within the phase II clinical trial portion of the study with a 1:1 randomization ratio.	• Frequency and severity of adverse events (AE) • Time to clearance of SARS-CoV-2	Not yet recruiting April 28, 2020	Phase 1 Phase 2
4.	Treatment of Severe COVID-19 Pneumonia With Allogeneic Mesenchymal Stromal Cells (COVID_MSV) (COVID_MSV) Sponsor: Red de Terapia Celular	NCT04361942	Valladolid, Spain	Double-blind, placebo-controlled, N:24, mesenchymal stromal cells, evaluate safety and efficacy of mesenchymal stromal cells from allogenic tissue for treatment of acute respiratory failure in patients with COVID-19.	• Proportion of patients who have achieved withdrawal of invasive mechanical ventilation • Rate of mortality	Recruiting April 24, 2020	Phase 2
5.	Safety and Effectiveness of Mesenchymal Stem Cells in the Treatment of Pneumonia of Coronavirus Disease 2019 Sponsor: Fuzhou General Hospital	NCT04371601	Fuzhou, Fujian, China	Open label, randomized, *N*: 60, control group: standard symptomatic treatments like antiviral (Oseltamivir), hormones, oxygen therapy, mechanical ventilation, and different accessory therapies; Experimental group: On the basis of the above-named standard symptomatic treatment and supportive medical aid, UC-MSCs were given at 106 / kg weight / time, once each 4 days for four times. iv infusion was given at intervals 3 days of 1st admission.	• Changes of oxygenation index (PaO2/FiO2),blood gas test	Active, not recruiting May 1, 2020	Early Phase 1
6.	Novel Adoptive Cellular Therapy With SARS-CoV-2 Specific T Cells in Patients With Severe COVID-19 Sponsor: KK Women’s and Children’s Hospital	NCT04351659	Singapore	Observational, novel adoptive cellular therapy with SARS-CoV-2-specific T cells in patients with severe COVID-19,N:8	• Success rate in production of SARS-CoV-2 specific T cells from convalescent donor	Recruiting April 17, 2020	….
7.	Bone Marrow-Derived Mesenchymal Stem Cell Treatment for Severe Patients With Coronavirus Disease 2019 (COVID-19) Sponsor: Guangzhou Institute of Respiratory Disease	NCT04346368	Guangzhou, Guangdong, China	Randomized controlled trial, parallel, *N* = 20, BM-MSCs in severe patients with coronavirus disease 19.	• Changes of oxygenation index (PaO2/FiO2) • Side effects in the BM-MSCs treatment group	Not yet recruiting April 15, 2020	Phase 1 Phase 2
8.	Mesenchymal Stromal Cells for the Treatment of SARS-CoV-2 Induced Acute Respiratory Failure (COVID-19 Disease) Sponsor: Baylor College of Medicine	NCT04345601	Houston, Texas, USA	Pilot study, *N* = 30,, BM-MSCs for the treatment of SARS-CoV-2 induced acute respiratory failure	• Incidence of unexpected adverse events • Improved oxygen saturations ≥93%	Not yet recruiting April 20, 2020	Early Phase 1
9.	Phase I / II Clinical Study of Immunotherapy Based on Adoptive Cell Transfer as a Therapeutic Alternative for Patients With COVID-19 in Colombia Sponsor: Universidad Nacional de Colombia	NCT04344548	Bogota, Cundinamarca, Colombia	Open label, single group assignment, *N* = 10, allogeneic NK transfer	• Adverse effects and safety	Not yet recruiting April 14, 2020	Phase 1 Phase 2
10.	NK Cells Treatment for COVID-19 Sponsor: Xinxiang medical university	NCT04280224	Xinxiang, Henan, China	Open label, randomized, *N* = 30, natural killer cells treatment in pneumonia patients infected with 2019 novel coronavirus	• Improvement of clinical symptoms including duration of fever • Improvement of clinical symptoms including respiratory frequency	Recruiting February 21, 2020	Phase 1
11.	Cell Therapy Using Umbilical Cord-derived Mesenchymal Stromal Cells in SARS-CoV-2-related ARDS (STROMA-CoV2) Sponsors: Assistance Publique - Hôpitaux de Paris	NCT04333368	Paris, France	Triple, randomized *N* = 60 patients, 20 patients will be cell-treated whereas the remaining 40 patients will be injected with a placebo in addition to the standard of care.	• Respiratory efficacy evaluated by the increase in PaO2/FiO2 ratio from baseline to day 7 in the experimental group compared with the placebo group [time frame: from baseline to day 7]	Not yet recruiting April 3, 2020	Phase 1 Phase 2
12.	ASC Therapy for Patients With Severe Respiratory COVID-19 (ASC COVID-19) Sponsors: Rigshospitalet, Denmark	NCT04341610	Copenhagen, Denmark	Double-blind placebo-controlled, randomized, *N* = 40 participants, allogeneic adipose-derived, mesenchymal stem cells or placebo will be injected to COVID-19 patients having severe pulmonary dysfunction.	• Changes in clinical critical treatment index [time frame: day 7 from randomization]	Not yet recruiting April 10, 2020	Phase 1 Phase 2
13.	Safety and Efficacy of CAStem for Severe COVID-19 Associated With/Without ARDS Sponsors: Chinese Academy of Sciences	NCT04331613	Beijing, China	Open label, single group, CAStem will be injected to severe COVID-19 associated with or without acute respiratory distress syndrome (ARDS), CAStem will be administered iv route.	• Adverse reaction (AE) and severe adverse reaction (SAE) • Frequency of adverse reaction (AE) and severe adverse reaction (SAE) within 28 days after treatment • Changes of lung imaging examinations • Evaluation by chest CT	Recruiting April 3, 2020	Phase 1 Phase 2
14.	A Phase I/II Study of Universal Off-the-shelf NKG2D-ACE2 CAR-NK Cells for Therapy of COVID-19 Sponsors: Chongqing Public Health Medical Center	NCT04324996	Chongqing, China	Interventional, quadruple,randomized, a phase I/II study of universal off-the-shelf NKG2D-ACE2 CAR-NK cells secreting IL15 super agonist and GM-CSF-neutralizing scFv for treatment of COVID-19	• Clinical response [time frame: up to 28 days] • Side effects in the treatment group [time frame: up to 28 days]	Recruiting, March 27, 2020	Phase 1 Phase 2
15.	Clinical Research of Human Mesenchymal Stem Cells in the Treatment of COVID-19 Pneumonia Sponsors: Puren Hospital Affiliated to Wuhan University of Science and Technology	NCT04339660	Wuhan, Hubei, China	Triple, randomized, *N* = 30 Human mesenchymal stem cells in the treatment of COVID-19 pneumonia	• The immune function (TNFα, IL-1β, IL-6, TGF-β, IL-8, PCT, CRP) • Blood oxygen saturation	Recruiting April 9, 2020	Phase 1 Phase 2
16.	Stem Cell Educator Therapy Treat the Viral Inflammation Caused by Severe Acute Respiratory Syndrome Coronavirus 2 Sponsor: Tianhe Stem Cell Biotechnologies Inc.	NCT04299152	?	Randomized = 20 Two-arm, partially masked, single center clinical study to assess the safety, feasibility, and efficacy of SCE therapy for the treatment of patients with SARS-CoV-2.	• Determine the number of COVID-19 patients who were unable to complete SCE therapy	Not yet recruiting March 6, 2020	Phase 2
17.	Treatment With Mesenchymal Stem Cells for Severe Corona Virus Disease 2019(COVID-19) Sponsors: Beijing 302 Hospital	NCT04288102	Hubei, China	Prospective, double-blind, multicenter, randomized trial *N* = 60 severe COVID-19 patients randomized 2:1 to 3 iv doses of mesenchymal stem cells (MSCs) or placebo (saline).	• Improvement time of clinical critical treatment index within 28 days • Side effects in the MSCs treatment group	Recruiting; August 31, 2020/December 31, 2020	Phase 2
18.	Therapy for Pneumonia Patients infected by 2019 Novel Coronavirus Sponsors: Puren Hospital Affiliated to Wuhan University of Science and Technology	NCT04293692	China, Hubei	Triple-blinded RCT. *N* = 48 with moderate to severe COVID-19 randomized to UC-MSCs or placebo	• Size of lesion area by chest imaging • Blood oxygen saturation	Recruiting; May 12,020/Feb 1, 2021	Not Applicable
19.	Clinical Trial for Human Mesenchymal Stem Cells in the Treatment of Severe Novel Coronavirus Pneumonia (COVID-19) Sponsor: Chinese PLA General Hospital	ChiCTR2000030138	Hainan, China	Randomized, double-blind, placebo-controlled trial *N* = 60 randomized to human umbilical cord mesenchymal stem cells (UC-MSC), or placebo in COVID-19.	• Clinical index	Not yet recruiting; From 2020-02-24 to 2020-05-31	Phase 2
20.	Mesenchymal Stem Cell Treatment for Pneumonia Patients Infected With 2019 Novel Coronavirus Sponsors: Beijing 302 Hospital	NCT04252118	China	Open-label, non-randomized intervention study *N* = 20 patients with COVID-19 Treatment: *N* = 10 treated with MSN *N* = 10 treated with conventional treatment.	• Size of lesion area by chest radiograph or CT (time frame day 28) • Side effects day (time frame day 180)	Recruiting; Dec 2020/December, 2021	Phase 1
21.	A Pilot Clinical Study on Inhalation of Mesenchymal Stem Cells Exosomes Treating Severe Novel Coronavirus Pneumonia Sponsors: Ruijin Hospital	NCT04276987	China	Open-label pilot study *N* = 30 with severe COVID-19, Single group assignment	• Adverse reactions • Time to clinical improvement (28 days)	Not yet recruiting; Estimated study completion: July 31, 2020	Phase 1
22.	Study of Human Umbilical Cord Mesenchymal Stem Cells in the Treatment of Novel Coronavirus Severe Pneumonia Sponsor: Wuhan Union Hospital, China	NCT04273646	China, Hubei	Open-label, randomized study *N* = 48 with severe COVID-19; Randomized to stem cell therapy or placebo	• Pneumonia severity index • Oxygenation index (PaO2/FiO2)	Not yet recruiting; June 302,020/Feb 15, 2022	Not Applicable
23.	Safety and efficacy of umbilical cord blood mononuclear cells conditioned medium in the treatment of severe and critically novel coronavirus pneumonia (COVID-19): a randomized controlled trial Sponsor: Xiangyang 1st People’s Hospital	ChiCTR2000029569	China, Hubei	Open label *N* = 30 with severe and critical COVID-19. Randomized to stem cell or conventional treatment.	• PSI	Not recruiting From 2020-02-05 to 2021-04-30	0
24.	Umbilical Cord(UC)-Derived Mesenchymal Stem Cells(MSCs) Treatment for the 2019-novel Coronavirus (nCOV) Pneumonia Sponsor: ZhiYong Peng	NCT04269525	China, Hubei	Open label *N* = 10, serious or critical COVID-19	• Oxygenation index day 14 • Partial arterial oxygen pressure (PaO2) / oxygen concentration (FiO2)	Recruiting; April 30, 2020/Sept 30, 2020	Phase 2
25.	Clinical Study for Human Menstrual Blood-Derived Stem Cells in the Treatment of Acute Novel Coronavirus Pneumonia (COVID-19) Sponsor: The First Affiliated Hospital, College of Medicine, Zhejiang University	ChiCTR2000029606	Zhejiang, China	Open-label, 5-arm study. Critically ill patients treated with stem cells, conventional treatment, artificial liver therapy, artificial liver therapy + stem cells, or conventional treatment	• Mortality in patients	Recruiting; From 2020-01-15 to 2022-12-31	0
26.	Canceled by the investigator Clinical Study for Umbilical Cord Blood Mononuclear Cells in the Treatment of Acute Novel Coronavirus Pneumonia (COVID-19) Sponsor: Guangzhou reborn health management consultation co., LTD	ChiCTR2000029812	Guangdong, China	Open label, *N* = 60 patients with COVID-19 randomized to umbilical cord blood mononuclear cells or conventional treatment	• Time to disease recovery	Not recruiting; From 2020-02-20 to 2021-02-20	0
27.	Clinical Study of Cord Blood NK Cells Combined with Cord Blood Mesenchymal Stem Cells in the Treatment of Acute Novel Coronavirus Pneumonia (COVID-19) Sponsor: Guangzhou Reborn Health Management Consultation Co., LTD	ChiCTR2000029817	Guangdong, china	Open label, *N* = 60 patients with COVID-19 randomized to high-dose NK cells, and mesenchymal stem cells, conventional dose NK cells and mesenchymal stem cells, or preventive dose NK cells and mesenchymal stem cells.	• Time to disease recovery	Not recruiting; From 2020-02-20 to 2021-02-20	0
28.	Canceled by the investigator Clinical Study for Umbilical Cord Blood Plasma in the Treatment of Acute Novel Coronavirus Pneumonia (COVID-19) Sponsor: Guangzhou reborn health management consultation co., LTD	ChiCTR2000029818	Guangdong, china	Open label, *N* = 60 patients with COVID-19 randomized to high-dose NK cells, and mesenchymal stem cells, conventional dose NK cells and mesenchymal stem cells, or preventive dose NK cells and mesenchymal stem cells.	• Time to disease recovery	Not recruiting; From 2020-02-20 to 2021-02-20	0
29.	Clinical trials of mesenchymal stem cells for the treatment of pneumonitis caused by novel coronavirus pneumonia (COVID-19) Sponsor: Institute of Basic Medical Sciences, Chinese Academy of Medical Sciences	ChiCTR2000029990	Beijing, Hubei, Shanghai	*N* = 120, severe COVID-19 randomized to MSCs or saline	• Improved respiratory system function (blood oxygen saturation) recovery time	Recruiting; From 2020-01-30 to 2020-03-31	Phase 1–2
30.	Umbilical cord Wharton’s Jelly derived mesenchymal stem cells in the treatment of severe novel coronavirus pneumonia (COVID-19) Sponsor: The Sixth Medical Center of PLA General Hospital	ChiCTR2000030088	Beijing, China	Type of study not stated. Blinding not stated *N* = 40 with critical COVID-19. Treatment: stem cells (*n* = 20) 40 ml saline (*n* = 20)	• The nucleic acid of the novel coronavirus is negative • CT scan of ground-glass shadow disappeared	Not yet recruiting; From 2020-03-01 to 2021-12-31	0
31.	Safety and effectiveness of human umbilical cord mesenchymal stem cells in the treatment of acute respiratory distress syndrome of severe novel coronavirus pneumonia (COVID-19) Sponsor: The First Affiliated Hospital of Nanchang University	ChiCTR2000030116	Jiangxi, China	*N* = 16 with critical COVID-19; different stem cell doses	• Time to leave ventilator on day 28 after receiving MSCs infusion	Recruiting; From 2020-02-01 to 2020-08-31	N/A
32.	Canceled by the investigator Clinical study of mesenchymal stem cells in treating severe novel coronavirus pneumonia (COVID-19) Sponsor: The First Affiliated Hospital of Nanchang University	ChiCTR2000030224	Hubei, China	Clinical study, open label Severe or critical COVID-19 patients; *N* = 32 stratified severity and randomized to stem cells or injection with saline	• Several primary endpoints—not specified	Not yet recruiting; From 2020-02-14 to 2020-05-31	N/A
33.	Umbilical cord mesenchymal stem cells (hUC-MSCs) in the treatment of high risk novel coronavirus pneumonia (COVID-19) patients Sponsor: Nanjing Second Hospital	ChiCTR2000030300	Jiangsu, China	A single-center, single-arm, prospective, open clinical study *N* = 9. UC-MSCs will be injected to COVID-19 patients.	• Time to disease recovery; • Exacerbation (transfer to RICU) time	Recruiting; From 2020-02-19 to 2021-02-20	Phase 1
34.	Stem Cell Educator Therapy Treat the Viral Inflammation Caused by Severe Acute Respiratory Syndrome Coronavirus 2 Sponsor: Tianhe Stem Cell Biotechnologies Inc.	NCT04299152	?	This is a prospective, two-arm, partially masked, single-center clinical study. *N* = 20 patients with SARS-CoV-2 undergoing either stem cell therapy or conventional treatment	• Determine the number of COVID-19 patients who were unable to complete SCE therapy • The feasibility will be evaluated by the number of COVID-19 patients who were unable to complete SCE therapy	Not yet recruiting; Nov 2020	Phase 2
35.	Novel Coronavirus Induced Severe Pneumonia Treated by Dental Pulp Mesenchymal Stem Cells Sponsor: CAR-T (Shanghai) Biotechnology Co., Ltd.	NCT04302519	?	Single-arm study *N* = 24. Patients with severe COVID-19 assigned to stem cell therapy.	• Disappear time of ground-glass shadow in the lungs [time frame: 14 days]	Not yet recruiting, July 2021	phase 1
36.	Treatment of COVID-19 Patients Using Wharton’s Jelly-Mesenchymal Stem Cells Sponsor: Stem Cells Arabia	NCT04313322	Jordan	Single-arm study *N* = 5 with COVID-19	• Improvement of clinical symptoms; • Adverse events; • Viral RNA	Recruiting. Sept 2020	Phase 1
37.	NestCell® Mesenchymal Stem Cell to Treat Patients With Severe COVID-19 Pneumonia (HOPE) Sponsor: Azidus Brazil	NCT04315987	Not stated	*N* = 24 patients	• Disappear time of ground-glass shadow in the lungs	Not yet recruiting. June 2020	Phase 1–2
38.	Study the effect of intravenous injection of dental pulp mesenchymal stem cells in treatment of patients with COVID-19 pneumonia Sponsor: Kerman University of Medical Sciences	IRCT20140911019125N6	Iran, Kerman	Clinical trial without control group, community based, not blinded, non-randomized controlled study. Dental pulp mesenchymal stem cells will be injected intravenously at one time.	• Pulmonary condition • Expression of nucleic acid of virus • Lymphocyte count • Patients clinical signs	Recruiting, 2020-04-04,	Phase 2
39.	Evaluation of the efficacy and safety of cord-derived mesenchymal stem cell transplantation in the treatment of COVID-19 Sponsor: MOM research and innovation center	IRCT20140528017891N8	Iran, Tehran	This study was a parallel randomized controlled clinical trial study design. The sample size of the study is 10 corona virus patients that will be assigned to intervention and control groups using simple randomization method.	• Death • Evaluation of Pneumonia Severity Index • Evaluation of oxygen supply index • C- Reactive protein • Pro-calcitonin • Lymphocyte count • Counting of CD3 +, CD4 + and CD8 + T cells • + CD4 + / CD8 ratio • Improve pneumonia evaluated by CT scan	Recruitment complete, 2020-03-24	Phase 3
40.	Mesenchymal stem cell utilization in reducing complications and enhancing pneumonia healing in patients infected with 2019-nCoV (phase I clinical trial) Sponsor: Bagheiatallah University of Medical Sciences	IRCT20200325046860N2	Iran, Tehran	Experimental: mesenchymal stem cell (MSC) treatment group conventional treatment plus MSC participants will receive conventional treatment plus 3 times of MSCs (7.0 × 10E7 MSCs intravenously at day 0, day 3, day 6), 5 patients, clinical trial phase I No intervention: conventional control group without MSC therapy but conventional treatment should be received.	• Respiratory function of patients	Recruitment complete, 2020-04-01	Phase 1
41.	A Comparison Study on Safety & Efficacy of Repeated Intravenous Infusion of Allogeneic Mesenchymal Stem Cell from Different Sources in ARDS Patients: A Randomized, Double Blind, Clinical Trial Phase II Sponsor: Bagheiatallah University of Medical Sciences	IRCT20080901001165N44	Iran, Tehran	A uncontrolled, parallel, double-blind, randomized, clinical trial, phase II 4 groups, 3 patients in each group, totally 12 patients 4 cell interventional groups 12 months follow-up	• Numbers of patients occurred any unexpected severe adverse events (including all-cause deaths)	Recruiting, 2020-03-29	Phase 1–2
42.	Mesenchymal Stem Cell Therapy for Acute Respiratory Distress Syndrome in Coronavirus Infection: A Phase 2–3 Clinical Trial Sponsor: Iranian academic center for education culture and research	IRCT20200217046526N2	Iran, Tehran	A controlled randomized clinical trial phase 2–3	• Adverse events assessment • Blood oxygen saturation	Recruiting, 2020-04-05	Phase 2–3
43.	Cell therapy in patients with coronavirus19 using mesenchymal stem cells Sponsor: Barekat Pharmaceutical Group	IRCT20190717044241N2	Iran, Tehran	One group with 10 uncontrolled patients, Phase I clinical trial	• Clinical response • partial arterial oxygen pressure (PaO2) & oxygen concentration (FiO2)	Recruiting, 2020-04-20	Phase 1

### Concussion and future perspective

Given the prevalence of COVID-19 and its complications such as cytokine storm, which is followed by ARDS and death of patients, finding a way to treat and improve the patients is of high importance [116]. As mentioned in this paper, there is no specific therapy for this virus and supportive therapies as well as non-specific antiviral drugs are mainly used for this purpose. Today, cell therapy is a modern method for treating a variety of diseases and several studies have been conducted in recent months to treat the SARS-CoV-2 virus using stem cells, suggesting the application of MSCs or immune cells such as NK cells [33, 117, 118]. According to research on MSC-based therapy, the safety and immunomodulatory role of MSCS in ARDS have been approved [82]. MSCs can secrete factors that improve the lung microenvironment, inhibit immune system overactivation, promote tissue repair, rejuvenate alveolar epithelial cells, inhibit pulmonary counteracting fibrosis, or improve function in damaged lung tissue because of SARS-CoV-2 infection [119, 120].

Many issues related to the application of MSCs, including the ideal dose and optimum timing of MSC delivery, should be further explored. In several animal models of human diseases, the use of secretory exosomes from MSCs has been claimed to mimic the beneficial effects of MSCs in antiviral therapy for influenza virus, reducing virus replication in lungs and virus-induced release of pro-inflammatory cytokines [121, 122]. Experimental studies and ongoing randomized trials will play an essential role in the clarification of the therapeutic potential of MSCs, which further our understanding of how MSCs interact with lung tissue infected by SARS-CoV-2.

## Data Availability

Not applicable.

## References

[1] Rothan HA, Byrareddy SNJJ (2020). The epidemiology and pathogenesis of coronavirus disease (COVID-19) outbreak.

[2] Sajadi MM, Habibzadeh P, Vintzileos A, Shokouhi S, Miralles-Wilhelm F, Amoroso AJAS (2020). Temperature and latitude analysis to predict potential spread and seasonality for COVID-19.

[3] Park M, Cook AR, Lim JT, Sun Y, Dickens BL. A Systematic Review of COVID-19 Epidemiology Based on Current Evidence. J Clin Med. 2020;9(4):967. 10.3390/jcm9040967. 10.3390/jcm9040967 PMC7231098 32244365

[4] Debnath M, Banerjee M, Berk MJTFJ (2020). Genetic gateways to COVID-19 infection: implications for risk, severity, and outcomes.

[5] Zu ZY, Jiang MD, Xu PP, Chen W, Ni QQ, Lu GM (2020). Coronavirus disease 2019 (COVID-19): a perspective from China.

[6] Wacharapluesadee S, Duengkae P, Rodpan A, Kaewpom T, Maneeorn P, Kanchanasaka B, et al. Diversity of coronavirus in bats from Eastern Thailand. 2015;12(1):57. 10.1186/s12985-015-0289-1 PMC4416284 25884446

[7] Prompetchara E, Ketloy C, Palaga TJAPJAI. Immune responses in COVID-19 and potential vaccines: Lessons learned from SARS and MERS epidemic. 2020;38(1):1–9. 10.12932/AP-200220-0772 32105090

[8] Huang C, Wang Y, Li X, Ren L, Zhao J, Hu Y (2020). Clinical features of patients infected with 2019 novel coronavirus in Wuhan, China. Lancet (London, England).

[9] Astuti I, Ysrafil. Severe Acute Respiratory Syndrome Coronavirus 2 (SARS-CoV-2): An overview of viral structure and host response. Diabetes Metab Syndr. 2020;14(4):407–12. 10.1016/j.dsx.2020.04.020. Epub 2020 Apr 18. PMID: 32335367; PMCID: PMC7165108. 10.1016/j.dsx.2020.04.020 PMC7165108 32335367

[10] Mehta P, McAuley DF, Brown M, Sanchez E, Tattersall RS, Manson JJJTL. COVID-19: consider cytokine storm syndromes and immunosuppression. 2020;395(10229):1033–4. 10.1016/S0140-6736(20)30628-0 PMC7270045 32192578

[11] Guo T, Fan Y, Chen M, Wu X, Zhang L, He T (2020). Cardiovascular implications of fatal outcomes of patients with coronavirus disease 2019 (COVID-19).

[12] NITULEScU GM, PAUNEScU H, MOScHOS SA, Petrakis D, Nitulescu G, Ion GND (2020). Comprehensive analysis of drugs to treat SARS-CoV-2 infection: mechanistic insights into current COVID-19 therapies.

[13] Zhang W, Zhao Y, Zhang F, Wang Q, Li T, Liu Z (2020). The use of anti-inflammatory drugs in the treatment of people with severe coronavirus disease 2019 (COVID-19): The Perspectives of clinical immunologists from China. Clin Immunol.

[14] Chen C, Zhang XR, Ju ZY, He WF (2020). Advances in the research of cytokine storm mechanism induced by Corona Virus Disease 2019 and the corresponding immunotherapies. Zhonghua Shao Shang Za Zhi.

[15] Ai J, Ketabchi N, Verdi J, Gheibi N, Khadem Haghighian H, Kavianpour M (2019). Mesenchymal stromal cells induce inhibitory effects on hepatocellular carcinoma through various signaling pathways. Cancer Cell Int.

[16] Li H, Shen S, Fu H, Wang Z, Li X, Sui X (2019). Immunomodulatory functions of mesenchymal stem cells in tissue engineering. Stem Cells Int.

[17] Han Y, Li X, Zhang Y, Han Y, Chang F, Ding J (2019). Mesenchymal stem cells for regenerative medicine. Cells.

[18] Leibacher J, Henschler R (2016). Biodistribution, migration and homing of systemically applied mesenchymal stem/stromal cells. Stem Cell Res Ther.

[19] Ullah M, Liu DD, Thakor AS (2019). Mesenchymal stromal cell homing: mechanisms and strategies for improvement. iScience.

[20] Ghaffari-Nazari HJJSCRM. The known molecules involved in MSC homing and migration. 2018;3:1–4.

[21] Ejtehadifar M, Shamsasenjan K, Movassaghpour A, Akbarzadehlaleh P, Dehdilani N, Abbasi P (2015). The effect of hypoxia on mesenchymal stem cell biology. Adv Pharm Bull.

[22] Walter J, Ware LB, Matthay MAJTLRM. Mesenchymal stem cells: mechanisms of potential therapeutic benefit in ARDS and sepsis. 2014;2(12):1016–26. 10.1016/S2213-2600(14)70217-6 25465643

[23] Prockop DJ, Oh JYJM (2012). Mesenchymal stem/stromal cells (MSCs): role as guardians of inflammation. Mol Ther.

[24] Zhang C, Wu Z, Li JW, Zhao H, Wang GQ. Cytokine release syndrome in severe COVID-19: interleukin-6 receptor antagonist tocilizumab may be the key to reduce mortality. Int J Antimicrob Agents. 2020;55(5):105954. 10.1016/j.ijantimicag.2020.105954. 10.1016/j.ijantimicag.2020.105954 PMC7118634 32234467

[25] Ai J, Ketabchi N, Verdi J, Gheibi N, Haghighian HK, Kavianpour MJCCI. Mesenchymal stromal cells induce inhibitory effects on hepatocellular carcinoma through various signaling pathways. 2019;19(1):1–13. 10.1186/s12935-019-1038-0 PMC6894473 31827403

[26] Yan X-I, Fu C-J, Chen L, Qin J-H, Zeng Q, Yuan H-F (2012). Mesenchymal stem cells from primary breast cancer tissue promote cancer proliferation and enhance mammosphere formation partially via EGF/EGFR/Akt pathway. Breast Cancer Res Treat.

[27] Arslan F, Lai RC, Smeets MB, Akeroyd L, Choo A, Aguor EN, et al. Mesenchymal stem cell-derived exosomes increase ATP levels, decrease oxidative stress and activate PI3K/Akt pathway to enhance myocardial viability and prevent adverse remodeling after myocardial ischemia/reperfusion injury. 2013;10(3):301–12. 10.1016/j.scr.2013.01.002 23399448

[28] Khakoo AY, Pati S, Anderson SA, Reid W, Elshal MF, Rovira II (2006). Human mesenchymal stem cells exert potent antitumorigenic effects in a model of Kaposi's sarcoma. J Exp Med.

[29] Sohni A, Verfaillie CMJS (2013). Mesenchymal stem cells migration homing and tracking.

[30] Follin B, Juhl M, Cohen S, Pedersen AE, Kastrup J, Ekblond AJTEPBR. Increased paracrine immunomodulatory potential of mesenchymal stromal cells in three-dimensional culture. 2016;22(4):322–9. 10.1089/ten.teb.2015.0532 PMC4964752 26861485

[31] Najar M, Raicevic G, Fayyad-Kazan H, Bron D, Toungouz M, Lagneaux LJC. Mesenchymal stromal cells and immunomodulation: a gathering of regulatory immune cells. 2016;18(2):160–71. 10.1016/j.jcyt.2015.10.011 26794710

[32] Chiossone L, Conte R, Spaggiari GM, Serra M, Romei C, Bellora F, et al. Mesenchymal stromal cells induce peculiar alternatively activated macrophages capable of dampening both innate and adaptive immune responses. 2016;34(7):1909–21. 10.1002/stem.2369 27015881

[33] Jeyaraman M, Somasundaram R, Anudeep TC, Ajay SS, Vinodh KV, Jain R, et al. Mesenchymal stem cells (mscs) as a novel therapeutic option for nCOVID-19—a review. 2020;9(2):20–35.

[34] Khoury M, Cuenca J, Cruz FF, Figueroa FE, Rocco PR, Weiss DJJERJ (2020). Current status of cell-based therapies for respiratory virus infections: applicability to COVID-19.

[35] Wu C, Chen X, Cai Y, Zhou X, Xu S, Huang H (2020). Risk factors associated with acute respiratory distress syndrome and death in patients with coronavirus disease 2019 pneumonia in Wuhan, China.

[36] Metcalfe SM (2020). Mesenchymal stem cells and management of COVID-19 pneumonia. Med Drug Discovery.

[37] Liu B, Li M, Zhou Z, Guan X, Xiang Y. Can we use interleukin-6 (IL-6) blockade for coronavirus disease 2019 (COVID-19)-induced cytokine release syndrome (CRS)? J Autoimmun. 2020:102452. 10.1016/j.jaut.2020.102452 PMC7151347 32291137

[38] Conti P, Ronconi G, Caraffa A, Gallenga CE, Ross R, Frydas I, Kritas SK. Induction of pro-inflammatory cytokines (IL-1 and IL-6) and lung inflammation by Coronavirus-19 (COVI-19 or SARS-CoV-2): anti-inflammatory strategies. J Biol Regul Homeost Agents. 2020;34(2):1. 10.23812/CONTI-E. 10.23812/CONTI-E 32171193

[39] Zhou F, Yu T, Du R, Fan G, Liu Y, Liu Z (2020). Clinical course and risk factors for mortality of adult inpatients with COVID-19 in Wuhan, China: a retrospective cohort study.

[40] Alzghari SK, Acuna VS. Supportive treatment with Tocilizumab for COVID-19: a systematic review. J Clin Virol. 2020:104380. 10.1016/j.jcv.2020.104380 PMC7194791 32353761

[41] Nauta AJ, Fibbe WE (2007). Immunomodulatory properties of mesenchymal stromal cells. Blood..

[42] Kean TJ, Lin P, Caplan AI, Dennis JEJS (2013). MSCs: delivery routes and engraftment, cell-targeting strategies, and immune modulation.

[43] Ostrand-Rosenberg S, Horn LA, Haile ST (2014). The programmed death-1 immune-suppressive pathway: barrier to antitumor immunity. J Immunol.

[44] Strasser A, Jost PJ, Nagata S (2009). The many roles of FAS receptor signaling in the immune system. Immunity.

[45] Khatri M, Richardson LA, Meulia T (2018). Mesenchymal stem cell-derived extracellular vesicles attenuate influenza virus-induced acute lung injury in a pig model. Stem Cell Res Ther.

[46] Waszak P, Alphonse R, Vadivel A, Ionescu L, Eaton F, Thébaud B (2012). Preconditioning enhances the paracrine effect of mesenchymal stem cells in preventing oxygen-induced neonatal lung injury in rats. Stem Cells Dev.

[47] Li Y, Xu J, Shi W, Chen C, Shao Y, Zhu L (2016). Mesenchymal stromal cell treatment prevents H9N2 avian influenza virus-induced acute lung injury in mice. Stem Cell Res Ther.

[48] Chan MCW, Kuok DIT, Leung CYH, Hui KPY, Valkenburg SA, Lau EHY (2016). Human mesenchymal stromal cells reduce influenza a H5N1-associated acute lung injury in vitro and in vivo. Proc Natl Acad Sci U S A.

[49] Curley GF, Jerkic M, Dixon S, Hogan G, Masterson C, O’Toole D (2017). Cryopreserved, xeno-free human umbilical cord mesenchymal stromal cells reduce lung injury severity and bacterial burden in rodent Escherichia coli–induced acute respiratory distress syndrome. Crit Care Med.

[50] Lee JW, Fang X, Krasnodembskaya A, Howard JP, Matthay MA (2011). Concise review: mesenchymal stem cells for acute lung injury: role of paracrine soluble factors. Stem Cells.

[51] Wysoczynki M, Khan A, Bolli R (2018). New paradigms in cell therapy: repeated dosing, intravenous delivery, immunomodulatory actions, and new cell types. Circ Res.

[52] Quaedackers ME, Baan CC, Weimar W, Hoogduijn MJ (2009). Cell contact interaction between adipose-derived stromal cells and Allo-activated T lymphocytes. Eur J Immunol.

[53] Van Den Akker F, Deddens J, Doevendans P, Sluijter J (2013). Cardiac stem cell therapy to modulate inflammation upon myocardial infarction. Biochim Biophys Acta (BBA) General Subjects.

[54] Di Nicola M, Carlo-Stella C, Magni M, Milanesi M, Longoni PD, Matteucci P (2002). Human bone marrow stromal cells suppress T-lymphocyte proliferation induced by cellular or nonspecific mitogenic stimuli. Blood J Am Soc Hematol.

[55] Choi H, Lee RH, Bazhanov N, Oh JY, Prockop DJ (2011). Anti-inflammatory protein TSG-6 secreted by activated MSCs attenuates zymosan-induced mouse peritonitis by decreasing TLR2/NF-κB signaling in resident macrophages. Blood J Am Soc Hematol.

[56] Oh JY, Roddy GW, Choi H, Lee RH, Ylöstalo JH, Rosa RH (2010). Anti-inflammatory protein TSG-6 reduces inflammatory damage to the cornea following chemical and mechanical injury. Proc Natl Acad Sci.

[57] Dyer DP, Thomson JM, Hermant A, Jowitt TA, Handel TM, Proudfoot AE (2014). TSG-6 inhibits neutrophil migration via direct interaction with the chemokine CXCL8. J Immunol.

[58] Liang B, Chen J, Li T, Wu H, Yang W, Li Y (2020). Clinical remission of a critically ill COVID-19 patient treated by human umbilical cord mesenchymal stem cells. ChinaXiv.

[59] Lim J-Y, Im K-I, Lee E-S, Kim N, Nam Y-S, Jeon Y-W (2016). Enhanced immunoregulation of mesenchymal stem cells by IL-10-producing type 1 regulatory T cells in collagen-induced arthritis. Sci Rep.

[60] Griffin MD, Elliman SJ, Cahill E, English K, Ceredig R, Ritter T (2013). Concise review: adult mesenchymal stromal cell therapy for inflammatory diseases: how well are we joining the dots?. Stem Cells.

[61] Wang L-T, Ting C-H, Yen M-L, Liu K-J, Sytwu H-K, Wu KK (2016). Human mesenchymal stem cells (MSCs) for treatment towards immune- and inflammation-mediated diseases: review of current clinical trials. J Biomed Sci.

[62] Harrell CR, Sadikot R, Pascual J, Fellabaum C, Jankovic MG, Jovicic NA-O, et al. Mesenchymal stem cell-based therapy of inflammatory lung diseases: current understanding and future perspectives. (1687-966X (Print)). 10.1155/2019/4236973 PMC6525794 31191672

[63] Connick P, Kolappan M Fau - Crawley C, Crawley C Fau - Webber DJ, Webber Dj Fau - Patani R, Patani R Fau - Michell AW, Michell Aw Fau - Du M-Q, et al. Autologous mesenchymal stem cells for the treatment of secondary progressive multiple sclerosis: an open-label phase 2a proof-of-concept study. (1474–4465 (Electronic)). 10.1016/S1474-4422(11)70305-2 PMC3279697 22236384

[64] Wilson JG, Liu KD, Zhuo H, Caballero L, McMillan M, Fang X, et al. Mesenchymal stem (stromal) cells for treatment of ARDS: a phase 1 clinical trial. (2213–2619 (Electronic)). 10.1016/S2213-2600(14)70291-7 PMC4297579 25529339

[65] Saleh M, Taher M, Sohrabpour AA, Vaezi AA, Nasiri Toosi M, Kavianpour M (2020). Perspective of placenta derived mesenchymal stem cells in acute liver failure. Cell Biosci.

[66] Inamdar AC, Inamdar AA (2013). Mesenchymal stem cell therapy in lung disorders: pathogenesis of lung diseases and mechanism of action of mesenchymal stem cell. Exp Lung Res.

[67] Walter J, Ware LB, Matthay MA. Mesenchymal stem cells: mechanisms of potential therapeutic benefit in ARDS and sepsis. (2213–2619 (Electronic)). 10.1016/S2213-2600(14)70217-6 25465643

[68] Anjos-Afonso F, Siapati EK, Bonnet D (2004). In vivo contribution of murine mesenchymal stem cells into multiple cell-types under minimal damage conditions. J Cell Sci.

[69] Armitage J, Tan DB, Troedson R, Young P, Lam K-V, Shaw K (2018). Mesenchymal stromal cell infusion modulates systemic immunological responses in stable COPD patients: a phase I pilot study. Eur Respir J.

[70] Zheng G, Huang L, Tong H, Shu Q, Hu Y, Ge M (2014). Treatment of acute respiratory distress syndrome with allogeneic adipose-derived mesenchymal stem cells: a randomized, placebo-controlled pilot study. Respir Res.

[71] Wilson JG, Liu KD, Zhuo H, Caballero L, McMillan M, Fang X (2015). Mesenchymal stem (stromal) cells for treatment of ARDS: a phase 1 clinical trial. Lancet Respir Med.

[72] Matthay MA, Calfee CS, Zhuo H, Thompson BT, Wilson JG, Levitt JE (2019). Treatment with allogeneic mesenchymal stromal cells for moderate to severe acute respiratory distress syndrome (START study): a randomised phase 2a safety trial. Lancet Respir Med.

[73] Vlachakis D, Karozou A, Kossida SJIr, treatment. 3D molecular modelling study of the H7N9 RNA-dependent RNA polymerase as an emerging pharmacological target. 2013;2013. 10.1155/2013/645348 PMC3800656 24187616

[74] Imai M, Watanabe T, Kiso M, Nakajima N, Yamayoshi S, Iwatsuki-Horimoto K, et al. A highly pathogenic avian H7N9 influenza virus isolated from a human is lethal in some ferrets infected via respiratory droplets. 2017;22(5):615–26. e8. 10.1016/j.chom.2017.09.008 PMC5721358 29056430

[75] Xu Z, Shi L, Wang Y, Zhang J, Huang L, Zhang C (2020). Pathological findings of COVID-19 associated with acute respiratory distress syndrome. Lancet Respir Med.

[76] Gao C, Wang Y, Gu X, Shen X, Zhou D, Zhou S, et al. Community-acquired pneumonia-China N. association between cardiac injury and mortality in hospitalized patients infected with Avian Influenza A (H7N9) virus. 2020;48(4):451–8. 10.1097/CCM.0000000000004207 PMC7098447 32205590

[77] Khoury M, Alcayaga-Miranda F, Illanes SE, Figueroa FE (2014). The promising potential of menstrual stem cells for antenatal diagnosis and cell therapy. Front Immunol.

[78] Chen L, Qu J, Cheng T, Chen X, Xiang C (2019). Menstrual blood-derived stem cells: toward therapeutic mechanisms, novel strategies, and future perspectives in the treatment of diseases. Stem Cell Res Ther.

[79] Chen L, Qu J, Xiang C (2019). The multi-functional roles of menstrual blood-derived stem cells in regenerative medicine. Stem Cell Res Ther.

[80] Leng Z, Zhu R, Hou W, Feng Y, Yang Y, Han Q (2020). Transplantation of ACE2-mesenchymal stem cells improves the outcome of patients with COVID-19 pneumonia. Aging Dis.

[81] Wang T, Chen R, Liu C, Liang W, Guan W, Tang R (2020). Attention should be paid to venous thromboembolism prophylaxis in the management of COVID-19. Lancet Haematol.

[82] Cruz FF, Rocco PRM (2019). Cell therapy for acute respiratory distress syndrome patients: the START study. J Thorac Dis.

[83] Khoury M, Rocco PRM, Phinney DG, Krampera M, Martin I, Viswanathan S, Nolta JA, LeBlanc K, Galipeau J, Weiss DJ. Cell-Based Therapies for COVID-19: Proper Clinical Investigations are Essential. Cytotherapy. 2020. 10.1016/j.jcyt.2020.04.089. Epub ahead of print. PMCID: PMC7163352. 10.1016/j.jcyt.2020.04.089 PMC7163352 32933835

[84] Khoury M, Cuenca J, Cruz FF, Figueroa FE, Rocco PRM, Weiss DJ. Current status of cell-based therapies for respiratory virus infections: applicability to COVID-19. Eur Respir J. 2020;55(6):2000858. 10.1183/13993003.00858-2020. PMID: 32265310; PMCID: PMC7144273. 10.1183/13993003.00858-2020 PMC7144273 32265310

[85] London AJ, Kimmelman J (2020). Against pandemic research exceptionalism. Science..

[86] Ji F, Li L, Li Z, Jin Y, Liu W. Mesenchymal stem cells as a potential treatment for critically ill patients with coronavirus disease 2019. Stem Cells Transl Med. 2020. 10.1002/sctm.20-0083 PMC7264790 32320535

[87] Stockman LJ, Bellamy R, Garner P (2006). SARS: systematic review of treatment effects. PLoS Med.

[88] Galipeau J, Sensébé L (2018). Mesenchymal stromal cells: clinical challenges and therapeutic opportunities. Cell Stem Cell.

[89] Weiss DJ, English K, Krasnodembskaya A, Isaza-Correa JM, Hawthorne IJ, Mahon BP (2019). The necrobiology of mesenchymal stromal cells affects therapeutic efficacy. Front Immunol.

[90] Abou-El-Enein M, Bauer G, Reinke P (2014). The business case for cell and gene therapies. Nat Biotechnol.

[91] Abou-El-Enein M, Elsanhoury A, Reinke P (2016). Overcoming challenges facing advanced therapies in the EU market. Cell Stem Cell.

[92] Sheridan C. First off-the-shelf mesenchymal stem cell therapy nears European approval. Nature Biotechnology. 2018;36(3):212–4. 10.1038/nbt0318-212a. 10.1038/nbt0318-212a 29509727

[93] Moll G, Ankrum JA, Kamhieh-Milz J, Bieback K, Ringdén O, Volk H-D (2019). Intravascular mesenchymal stromal/stem cell therapy product diversification: time for new clinical guidelines. Trends Mol Med.

[94] Moll G, Alm JJ, Davies LC, von Bahr L, Heldring N, Stenbeck-Funke L (2014). Do cryopreserved mesenchymal stromal cells display impaired immunomodulatory and therapeutic properties?. Stem Cells.

[95] Moll G, Geißler S, Catar R, Ignatowicz L, Hoogduijn MJ, Strunk D, Bieback K, Ringdén O. Cryopreserved or Fresh Mesenchymal Stromal Cells: Only a Matter of Taste or Key to Unleash the Full Clinical Potential of MSC Therapy? Adv Exp Med Biol. 2016;951:77–98. 10.1007/978-3-319-45457-3_7. PMID: 27837556. 10.1007/978-3-319-45457-3_7 27837556

[96] Giri J, Galipeau J (2020). Mesenchymal stromal cell therapeutic potency is dependent upon viability, route of delivery, and immune match. Blood Adv.

[97] Moll G, Drzeniek N, Kamhieh-Milz J, Geissler S, Volk H-D, Reinke P (2020). MSC therapies for COVID-19: importance of patient coagulopathy, thromboprophylaxis, cell product quality and mode of delivery for treatment safety and efficacy. Front Immunol.

[98] Verdecchia P, Cavallini C, Spanevello A, Angeli FJEJIM (2020). The pivotal link between ACE2 deficiency and SARS-CoV-2 infection.

[99] Hamming I, Timens W, Bulthuis M, Lely A. Navis Gv, van Goor HJTJoPAJotPSoGB, et al. tissue distribution of ACE2 protein, the functional receptor for SARS coronavirus. A first step in understanding SARS pathogenesis. 2004;203(2):631–7. 10.1002/path.1570 PMC7167720 15141377

[100] Barkauskas CE, Cronce MJ, Rackley CR, Bowie EJ, Keene DR, Stripp BR, et al. Type 2 alveolar cells are stem cells in adult lung. 2013;123(7):3025–36. 10.1172/JCI68782 PMC3696553 23921127

[101] Rajaei S, Dabbagh A. The immunologic basis of COVID-19: a clinical approach. 2020. 2020;5(1):6 %J Journal of Cellular &amp; Molecular Anesthesia.

[102] Qi J, Zhou Y, Hua J, Zhang L, Bian J, Liu B, et al. The scRNA-seq expression profiling of the receptor ACE2 and the cellular protease TMPRSS2 reveals human organs susceptible to COVID-19 infection. bioRxiv. 2020; 2020.04.16.045690. 10.3390/ijerph18010284 PMC7794913 33401657

[103] Zhang H, Penninger JM, Li Y, Zhong N, Slutsky ASJI. Angiotensin-converting enzyme 2 (ACE2) as a SARS-CoV-2 receptor: molecular mechanisms and potential therapeutic target. 2020;46(4):586–90. 10.1007/s00134-020-05985-9 PMC7079879 32125455

[104] Lu N, Yang Y, Wang Y, Liu Y, Fu G, Chen D, et al. ACE2 gene polymorphism and essential hypertension: an updated meta-analysis involving 11,051 subjects. 2012;39(6):6581–9. 10.1007/s11033-012-1487-1 22297693

[105] Patel VB, Bodiga S, Basu R, Das SK, Wang W, Wang Z, et al. Loss of angiotensin-converting enzyme-2 exacerbates diabetic cardiovascular complications and leads to systolic and vascular dysfunction: a critical role of the angiotensin II/AT1 receptor axis. 2012;110(10):1322–35. 10.1161/CIRCRESAHA.112.268029 PMC3746191 22474255

[106] Devaux CA, Rolain J-M, Raoult DJJoM, Immunology, Infection. ACE2 receptor polymorphism: Susceptibility to SARS-CoV-2, hypertension, multi-organ failure, and COVID-19 disease outcome. 2020. 10.1016/j.jmii.2020.04.015 PMC7201239 32414646

[107] Reddy Gaddam R, Chambers S, Bhatia MJI, Targets A-D. ACE and ACE2 in inflammation: a tale of two enzymes. 2014;13(4):224–34. 10.2174/1871528113666140713164506 25019157

[108] Thomas MC, Pickering RJ, Tsorotes D, Koitka A, Sheehy K, Bernardi S, et al. Genetic Ace2 deficiency accentuates vascular inflammation and atherosclerosis in the ApoE knockout mouse. 2010;107(7):888–97. 10.1161/CIRCRESAHA.110.219279 20671240

[109] Li M-Y, Li L, Zhang Y, Wang X-SJI. Expression of the SARS-CoV-2 cell receptor gene ACE2 in a wide variety of human tissues. 2020;9:1–7. 10.1186/s40249-020-00662-x PMC7186534 32345362

[110] Geng Y-J, Wei Z-Y, Qian H-Y, Huang J, Lodato R, Castriotta RJ. Pathophysiological characteristics and therapeutic approaches for pulmonary injury and cardiovascular complications of coronavirus disease 2019. Cardiovasc Pathol. 2020:107228. 10.1016/j.carpath.2020.107228 PMC7162778 32375085

[111] Lee RH, Pulin AA, Seo MJ, Kota DJ, Ylostalo J, Larson BL (2009). Intravenous hMSCs improve myocardial infarction in mice because cells embolized in lung are activated to secrete the anti-inflammatory protein TSG-6. Cell Stem Cell.

[112] Shetty AK, Upadhya R, Madhu LN, Kodali M (2019). Novel insights on systemic and brain aging, stroke, amyotrophic lateral sclerosis, and Alzheimer’s disease. Aging Dis.

[113] Shetty AK, Kodali M, Upadhya R, Madhu LN (2018). Emerging anti-aging strategies-scientific basis and efficacy. Aging Dis.

[114] Thomas R, Wang W, Su D-M (2020). Contributions of age-related thymic involution to Immunosenescence and Inflammaging. Immun Ageing.

[115] Shetty AK (2020). Mesenchymal stem cell infusion shows promise for combating coronavirus (COVID-19)-induced pneumonia. Aging Dis.

[116] Yi Y, Lagniton PNP, Ye S, Li E, Xu R-H (2020). COVID-19: what has been learned and to be learned about the novel coronavirus disease. Int J Biol Sci.

[117] Monteil V, Kwon H, Prado P, Hagelkrüys A, Wimmer RA, Stahl M (2020). Inhibition of SARS-CoV-2 infections in engineered human tissues using clinical-grade soluble human ACE2.

[118] Ji H, Yan Y, Ding B, Guo W, Brunswick M, Niethammer A (2020). Novel decoy cellular vaccine strategy utilizing transgenic antigen-expressing cells as immune presenter and adjuvant in vaccine prototype against SARS-CoV-2 virus.

[119] Foronjy RF, Majka SM (2012). The potential for resident lung mesenchymal stem cells to promote functional tissue regeneration: understanding microenvironmental cues. Cells.

[120] Golchin A, Seyedjafari E, Ardeshirylajimi A. Mesenchymal stem cell therapy for COVID-19: present or future. Stem Cell Rev Rep. 2020:1–7. 10.1007/s12015-020-09973-w PMC7152513 32281052

[121] Khatri M, Richardson LA, Meulia T (2018). Mesenchymal stem cell-derived extracellular vesicles attenuate influenza virus-induced acute lung injury in a pig model. Stem Cell Res Ther.

[122] Bruno S, Deregibus MC, Camussi G (2015). The secretome of mesenchymal stromal cells: role of extracellular vesicles in immunomodulation. Immunol Lett.

